# Learning and executing goal-directed choices by internally generated sequences in spiking neural circuits

**DOI:** 10.1371/journal.pcbi.1005669

**Published:** 2017-07-31

**Authors:** John Palmer, Adam Keane, Pulin Gong

**Affiliations:** 1 School of Physics, University of Sydney, Sydney, NSW, Australia; 2 Centre of Excellence for Integrative Brain Function, University of Sydney, Sydney, NSW, Australia; Brain and Spine Institute (ICM), FRANCE

## Abstract

Recent neural ensemble recordings have established a link between goal-directed spatial decision making and internally generated neural sequences in the hippocampus of rats. To elucidate the synaptic mechanisms of these sequences underlying spatial decision making processes, we develop and investigate a spiking neural circuit model endowed with a combination of two synaptic plasticity mechanisms including spike-timing dependent plasticity (STDP) and synaptic scaling. In this model, the interplay of the combined synaptic plasticity mechanisms and network dynamics gives rise to neural sequences which propagate ahead of the animals’ decision point to reach goal locations. The dynamical properties of these forward-sweeping sequences and the rates of correct binary choices executed by these sequences are quantitatively consistent with experimental observations; this consistency, however, is lost in our model when only one of STDP or synaptic scaling is included. We further demonstrate that such sequence-based decision making in our network model can adaptively respond to time-varying and probabilistic associations of cues and goal locations, and that our model performs as well as an optimal Kalman filter model. Our results thus suggest that the combination of plasticity phenomena on different timescales provides a candidate mechanism for forming internally generated neural sequences and for implementing adaptive spatial decision making.

## Author summary

Adaptive goal-directed decision making is critical for animals, robots and humans to navigate through space. In this study, we propose a novel neural mechanism for implementing spatial decision making in cued-choice tasks. We show that in a spiking neural circuit model, the interplay of network dynamics and a combination of two synaptic plasticity rules, STDP and synaptic scaling, gives rise to neural sequences. When a model rat pauses around a decision point, these sequences propagate ahead of the animal’s current location and travel towards a goal location. The dynamical properties of these forward-sweeping sequences and the rate of correct responses made by them are consistent with experimental data. In addition, we demonstrate that STDP when complemented by slower synaptic scaling enables neural sequences to make adaptive choices under probabilistic and time-varying cue-goal associations. The adaptive performance of our sequence-based network is comparable to a mathematical model, namely the Kalman filter, which is optimal for this adaptive task. Our results thus shed new light on our understanding of neural mechanisms underlying goal-directed decision making.

## Introduction

Neural sequences have been widely observed in many brain areas including the cortex [1, 2, 3, 4], and the hippocampus [5, 6, 7, 8, 9, 10, 11]. Based on how sequences are initialized, they can be placed into two broad categories, namely externally and internally generated sequences [12]. Externally generated sequences (EGS) are those which directly reflect an ongoing behavioural sequence such as actions [13] or positions visited [12, 14]; whilst internally generated sequences (IGS) arise either spontaneously or by being triggered by non-sequential external cues [12]. IGS have been argued to underlie predictions [15], goal-directed planning and decision making [6, 12, 16, 17].

One area where IGS have been extensively examined is the rodent hippocampus during navigational tasks [6, 18, 11, 15]. Most of these tasks follow a similar basic procedure; rodents are introduced to a maze and must navigate towards goal locations [6, 18, 15]. Recent experimental studies with multi-electrode array recordings have revealed that when the animals rest between goal-directed spatial navigation episodes, neural ensemble activity propagates forward towards potential goal locations [15]. Such recordings of rodents trained on spatial decision tasks have also found that when rodents paused around the decision point, forward sweeping IGS were formed [6]. Reconstructed locations from these IGS were found predominately forward of the animal’s position, indicating that these IGS are related to representation of future paths rather than pinpointing the current location or being a replay of recent history. Furthermore, the IGS appears to be used for making a goal-related choice, as the path chosen by the animal through the T-maze was strongly correlated with the path reconstructed from the IGS. Despite the importance of IGS for goal-directed behaviours such as spatial decision making, the neural mechanism underlying the formation of these IGS and their general computational roles remain unclear.

To address these issues, we build a spiking neural circuit model endowed with a combination of STDP and synaptic scaling, and show that the model is able to reproduce the dynamical properties of IGS and the behavioural response of correct rates of binary choices as reported in [6]. As in previous modelling studies [19], STDP in our model can learn the paths taken by moving rodents. Synaptic scaling, however, can prevent a positive feedback loop caused by STDP, and provides a separation of temporal scales needed for adaptive choice under uncertainty. We show that STDP complemented with slower homeostatic synaptic scaling is necessary to account for the properties of forward sweeping IGS recorded in [6], thus unravelling a mechanism for IGS propagation in the spatial decision making circuit.

To further study the general computational role of IGS in spatial decision making, we go beyond the deterministic association of cue and goal as used in [6], considering cases where the association between cue and goal is stochastic and varies over time. For these cases, our results are primarily focused on correct decisions on a trial basis; we find that the correct choice made by the model based on IGS can effectively track the time-varying cue-goal association, and that this process can be described as a recursive probabilistic inference. We show that the performance of this inference process implemented by our spatial decision making neural circuit is comparable to that of a Kalman filter [20], which is optimal for the cases we consider. In our model, the interplay of spiking sequences and the combined synaptic plasticity rules lead to constant changes of synaptic strengths which are proportional to the probability of cue-goal association. These changes can serve as a posterior for test trials which can then be exploited by IGS to make a choice; the IGS, combined with plasticity mechanisms, are essential for implementing this optimal inference. Our model offers an explicit formal characterization of probabilistic inference involved in the IGS-forming spiking neural circuit, thus establishing a link between such an inference and spatial decision making [12, 21].

## Results

### T-maze spatial decision tasks

**Fig 1:**
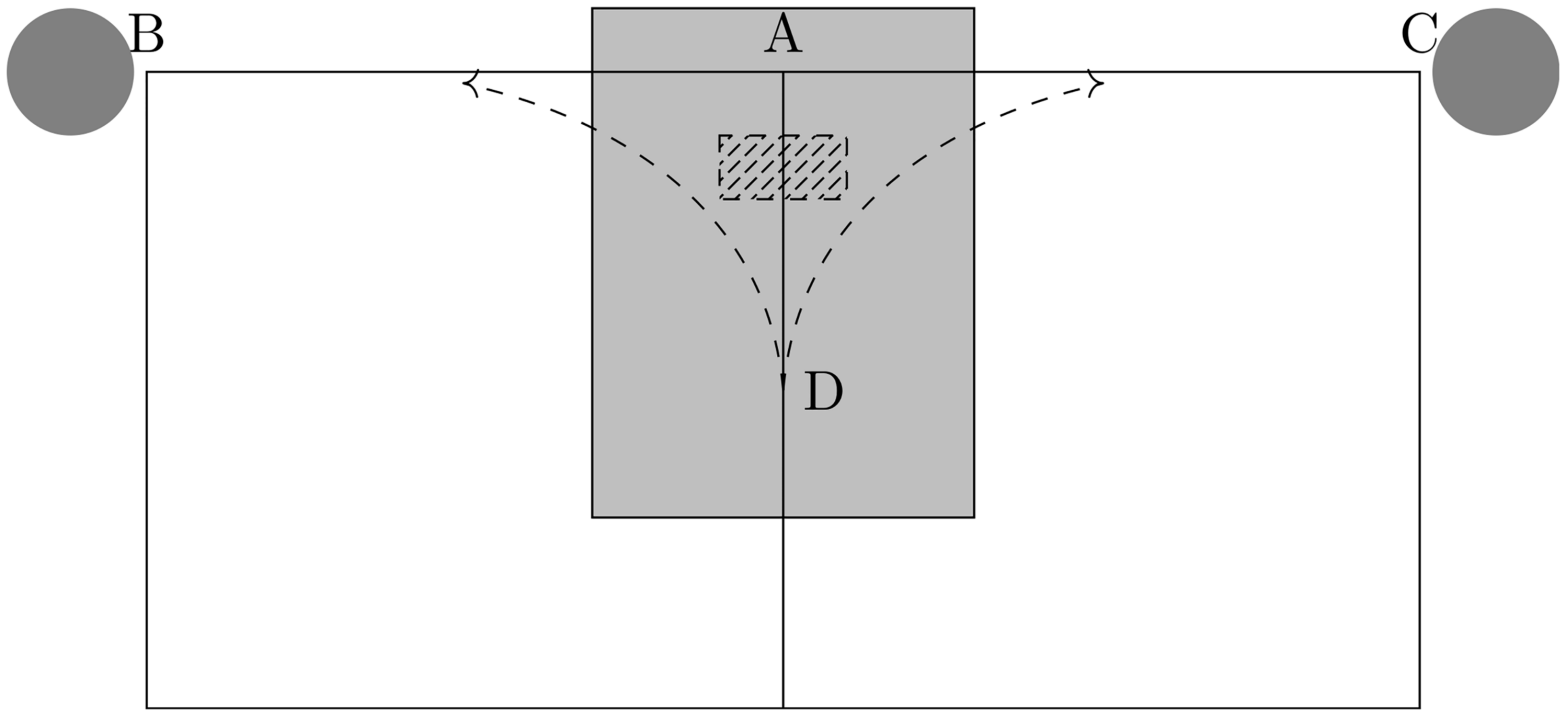
Schematic of the cued-choice task in a T-maze. A rat starts at D and then travels towards the decision point, A. When it enters the region marked by the hatched rectangle, a sound cue is played. Depending on the frequency of the cue, the goal is located at either B or C, indicated with grey circles. The rat then returns to D, when the next trial starts. The decision modelled in our neural circuit occurs in the shaded rectangle.

We begin by briefly describing the T-maze based decision task used in [6], which we aim to reproduce in a spiking neural circuit. In this task, rats were made to run laps through a T-maze, augmented with return arms, as shown in Fig 1. When the rats approached the decision point of the T-maze (A in Fig 1), one of two sound cues was played. These two cues were differentiated by their frequencies, and provided information to the rats about the location of a reward within the T-maze. For example, when the low or high-frequency cues were applied, the reward was on the left or right arm of the T-maze, respectively. Training began with a directed pre-training phase, in which the rats were prevented from choosing the incorrect arm of the T-maze. Training then continued following a similar procedure, except that the rats were free to travel down the incorrect arm of the T-maze. The rate at which the rats correctly identified the rewarded arm corresponding to the applied cue was monitored during this second training phase, and the activity of neural ensembles from the CA3 region was recorded. Analysis of the neural activity revealed a transient, but repeatable, phenomenon: as a rat approached the decision point, the neural representation of the rat’s location swept forward, creating a sequence of neural activity. These sequences were coherent and preferentially swept ahead of the animal rather than behind, implying that they represented future choices rather than recently travelled paths [6, 12].

We use a two-dimensional spiking neural circuit to reproduce the forward-sweeping sequences observed in [6]. In our model, we consider synaptic plasticity dynamics, which are determined by a combination of STDP and synaptic scaling. The external cues are applied by activating the cue neurons, which are randomly coupled to the other neurons in the two-dimensional network. For a full description of our neural circuit model, see Materials and methods. To investigate the neural mechanisms of forward-sweeping neural sequences and their functional roles in learning and implementing goal-directed choice, we apply the same task and training protocol as in [6].

During training, when a model rat moves along a T-maze, as in Fig 1, a localized activity pattern surrounding its position is initialized, corresponding to the behaviour of place fields in the hippocampus [22]. As the model rat approaches the decision point of the T-maze, one of a pair of cues is activated. The cue corresponding to the low-frequency sound signal is denoted by *C*1 and that for the high-frequency by *C*2, with *C*1 and *C*2 indicating that the goal is located on the left and right arm of the T-maze, respectively. As the model rat is restricted to move along the T-maze, we similarly restrict neurons from firing if their place fields are outside the T-maze.

**Fig 2:**
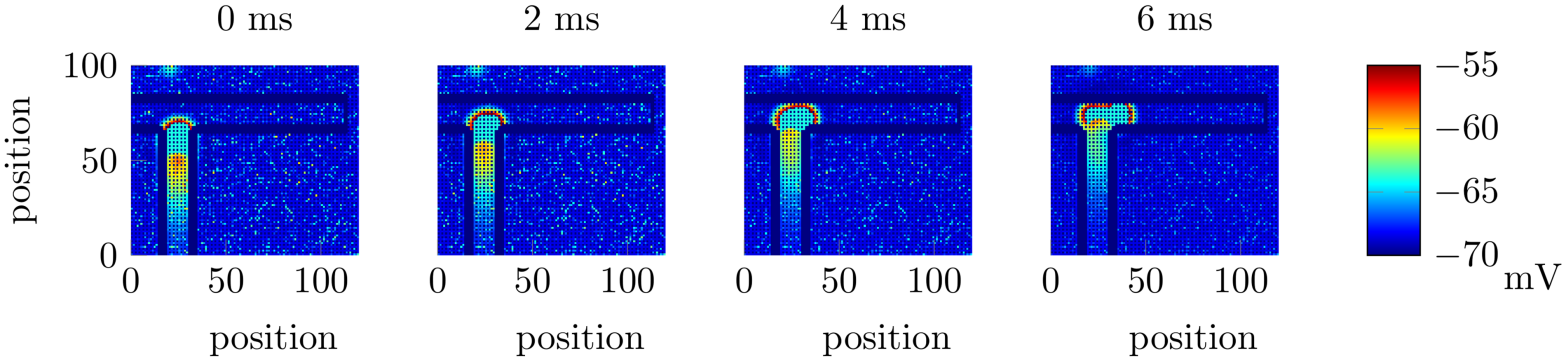
Forward-sweeping neural sequences at the decision point. Each frame is 2 ms after the previous one during a test trial and shows a snapshot of the membrane potentials of the excitatory neurons from a region of the network near the intersection of the T-maze. These snapshots are taken from the network with synaptic scaling, although without synaptic scaling, similar behaviour is seen on successful trials, but the proportion of successful trials is reduced. Neurons are located at integer locations along both axes.

For a training trial in our neural circuit model, as in [6], one arm of the T-maze is blocked. This is implemented by fixing the potential of the neurons in one arm of the T-maze; this forces the EGS to travel down the non-blocked arm. For instance, when the cue *C*1 is applied, the right arm of the T-maze is blocked, i.e., the model rat can only take the left arm. On each training trial, the sequence reaches the goal location, i.e., the end of the arm. After each training trial, a test trial is run, in which neither arm of the T-maze is blocked. By running a test trial after each training trial, it is possible to monitor in detail the behaviour of the neural circuit during the training process. For a test trial, the cue is activated as the model rat approaches the decision point. Under this protocol, if a forward-sweep sequence is formed and then travels down the arm of the T-maze corresponding to the goal location associated with the applied cue, whilst not reaching the goal location associated with the other cue, a successful choice is made by the network. In our scheme, we do not model reward explicitly. Fig 2 shows such a forward-sweeping sequence shortly after the activation of the cue *C*2; this sequence in not related to the sequential movement of the model rat, so it is an IGS [12]. Note that the EGS is similar to the IGS except that the EGS is achieved by forcing the sequence to travel along a specific path.

For the training trials, both STDP and synaptic scaling are enabled, whilst test trials are run with both synaptic rules disabled to prevent the test trials from affecting the training process. As in [6], the number of trials with the goal on the left and right is equal; this is achieved by alternating between *C*1 and *C*2 on successive training trials.

### Goal directed choices executed by IGS

We now demonstrate that our model is able to reproduce forward-sweeping neural sequences as observed in [6], and that such sequences can be used to make a correct choice of the goal location based on an external cue. In experimental studies, multiple animals were typically used to obtain average response rates [6, 18, 11]. In a similar way, we use multiple realizations of our neural circuit model and track average response rates during the training process. For each realization of the neural circuit, the connections between the cue neurons and the neurons with the 2D network and their coupling weights are randomly selected.

**Fig 3:**
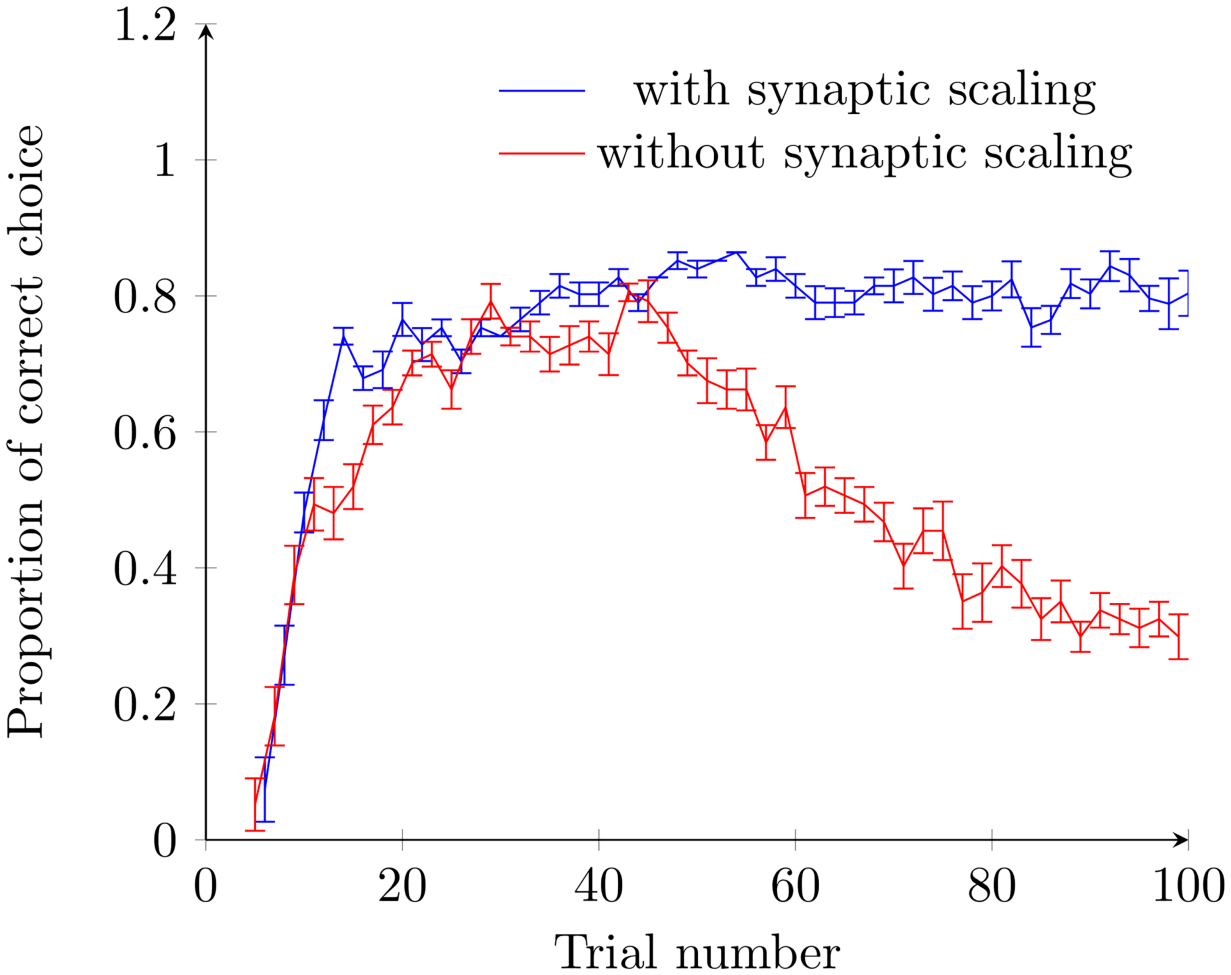
Proportion of correct choice versus trial number. In this case, cue *C*2 is always applied as the test cue. The blue curve shows the proportion of correct choices for the spiking neural circuit model with both STDP and synaptic scaling, whilst the red curve shows the neural circuit model without synaptic scaling. The bars show the standard error of the mean. Initially, both models show similar behaviour, namely an initial proportion of correct choice rate which is very low. After continued training, the accuracy of the model without synaptic scaling then begins to decrease. This decrease is due to an increase in the proportion of neural circuits in which the propagating wave pattern splits and travels along both arms of the T-maze. This decrease in accuracy is not observed in the full model, which maintains accuracy even as training continues. The final success rate of 80% is similar to that observed in experimental studies (75% in [6]).

As shown in Fig 3, in our models, the proportion of correct responses increases rapidly during the initial period of the training process. It eventually saturates at a threshold which is close to, but less than 1, meaning that even after extensive training, the neural circuit does not always make a correct choice; in other words, the IGS does not always travel to the goal location associated with the cue. In experimental studies in which rats learn to navigate a maze under a similar training paradigm, the rate at which rats correctly identify the rewarded arm of a T-maze shows similar behaviour, with an initial low correct response rate which increases before saturating at a level slightly under 1 (see Figure 4 in [6]). There are three possible ways in which the neural circuit fails to makes a correct choice of the goal location. In the first failure type, the sequence fails to propagate down either arm of the T-maze; this failure occurs most commonly in the early trials because the model rat has not learned the environment, in particular the goal location. For the second failure type, there are sequences propagating down both arms of the T-maze simultaneously, indicating that the network has been unable to make a correct choice about the goal location; this failure mode only occurs rarely in our model. In [6], it was found that the place fields of cells involved in an IGS tended to belong to one arm or the other, and IGS which activated neurons corresponding to both arms was rare. Finally, the sequence may simply propagate along the wrong arm of the T-maze; in experimental studies this failure type has been reported and has been correlated with the animal choosing the non-rewarded arm of the T-maze [6].

### Formation mechanisms of IGS

**Fig 4:**
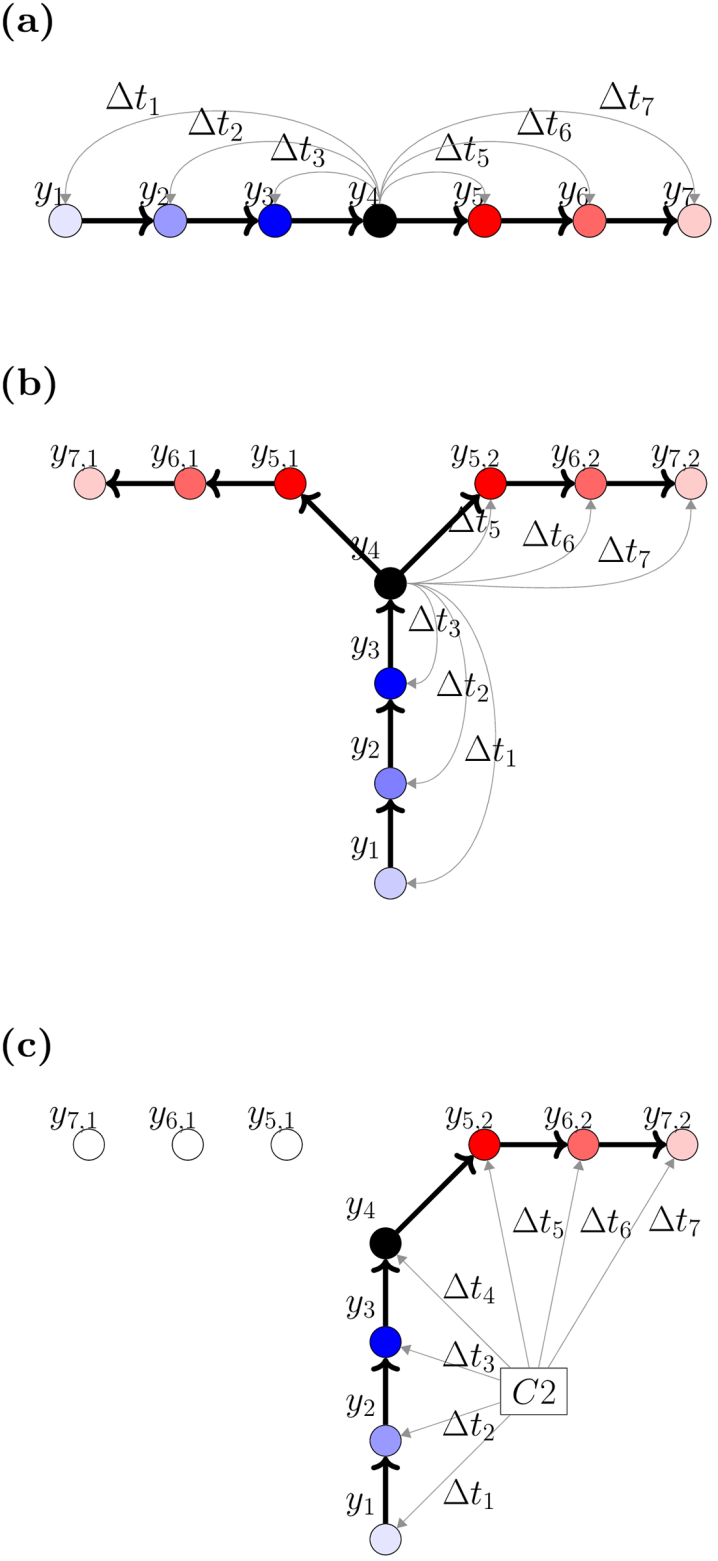
Schematic illustration of the changes of coupling strengths caused by STDP. Connection strengths from the black neuron to the neurons in blue are decreased, whilst those in red are increased. Color intensity is proportional to the magnitudes of the changes in coupling strengths. Thick arrows show the path of a neural sequence, and thin grey arrows show the connections of interest. (a) The effect of propagation of a sequence and STDP on recurrent connections. Connections in the direction of propagation of the sequence are increased, whilst those in the reverse direction are decreased. (b) The effect of training in the T-maze on recurrent connections in the T-maze. Connection strengths from the center stem to neurons along the left and right arm are increased equally due to the symmetry in the training process which has an equal number of training trials along each arm of the T-maze. (c) The effect of STDP on the connections from the external cue neuron *C*2 to the other neurons in the spiking neural circuit. The cue is activated when the sequence is located at neuron *y*_4_. The behaviour is similar to (b); however, only connection strengths along one path are increased, breaking the symmetry in connection strengths as shown in (b).

We now consider how forward-sweeping IGS can emerge from the network and why they can generate correct responses as shown in Figs 2 and 3. To this end, we consider a simple case, in which a spiking sequence is evoked by a model rat moving along a straight line, as shown in Fig 4a; i.e. neurons *y*_1_, *y*_2_, …, *y*_7_ are activated sequentially. Without loss of generality, we use a representative neuron, *y*_4_, to study the change of coupling strength to the other neurons due to the interplay between this evoked sequence and STDP. Later, we will discuss the effect of synaptic scaling. We let Δ*t*_*i*_ be the time between when neuron *y*_*i*_ spikes and when our reference neuron (*y*_4_) spikes. Based on the propagation of the evoked spiking sequence, it is clear that Δ*t*_1_ < Δ*t*_2_ < Δ*t*_3_ < Δ*t*_4_ = 0 < Δ*t*_5_ < Δ*t*_6_ < Δ*t*_7_. The change in coupling strengths, Δ*W*_*i*_, from *y*_4_ to the other neurons can be calculated by using the STDP window function (see Eq 22 in Materials and methods):

$$\begin{array}{c} \Delta W_{i}=\mathrm{sgn}\left(\Delta t_{i}\right)A\exp(-|\Delta t_{i}\left|/\tau \right), \end{array}$$ (1)

where the sign function, sgn(*x*) = −1 if *x* < 0, sgn(0) = 0 and sgn(*x*) = 1 if *x* > 0. It follows that Δ*W*_5_ > Δ*W*_6_ > Δ*W*_7_ > Δ*W*_4_ = 0 > Δ*W*_1_ > Δ*W*_2_ > Δ*W*_3_. In other words, connection strengths in the direction in which the model rat moves are increased while those in the reverse direction are decreased. Suppose then that this procedure is repeated many times, so that the path taken by the model rat is learned by the network: when neuron *y*_4_ is activated again, due to the directionality of the coupling strength changes, the membrane potential of neuron *y*_5_ will be greater than *y*_3_, and so on.

**Fig 5:**
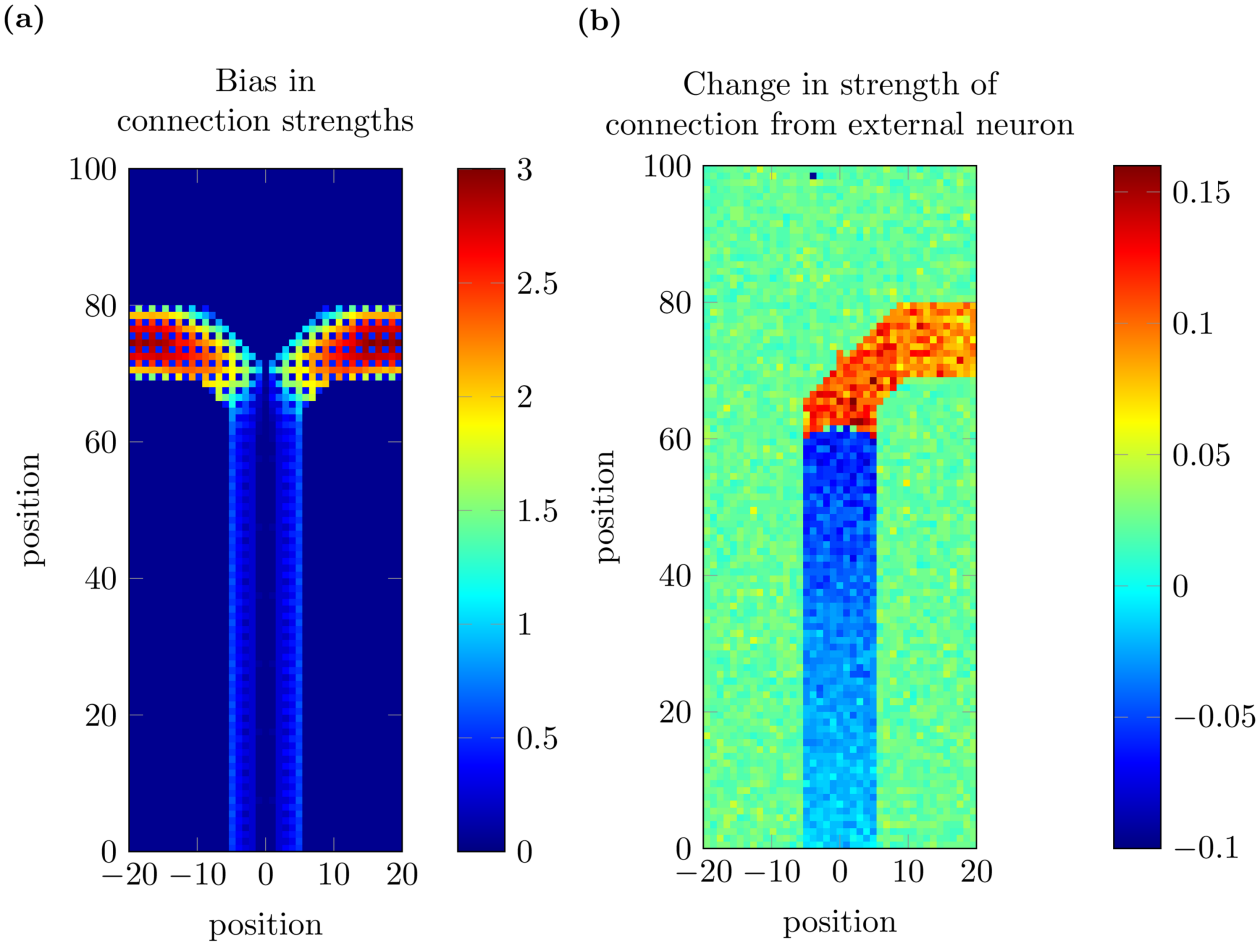
Changes to connection strengths after 20 training trials in the spiking neural circuit model. Coordinates of the x-axis are centered on the center stem to more clearly show the level of symmetry present in connection strength changes. We note that after 20 trials, these values are quite stable. (a) The bias (calculated using Eq 27) in connections from neurons in the center stem to other neurons in the 2D spiking neural circuit is small. For neurons on the two arms, the values are close to the theoretical maximum value, slightly larger than 3. (b) Changes to coupling strength from the external cue neuron are not symmetric. The average change in coupling strength for connections from the external cue neuron, *C*2 in this case, shows a clear bias towards the right hand side of the T-maze.

Now, consider a group of neurons arranged as in the decision making task shown in Figs 1 and 4b: neurons *y*_1_, …, *y*_4_ are placed along the center stem of the T-maze, while neurons *y*_5,1_, *y*_6,1_, *y*_7,1_ are along the left arm, and *y*_5,2_, *y*_6,2_, *y*_7,2_ are along the right arm. As outlined earlier, an equal number of training trials go along each arm. As a result, the connections from *y*_4_ to *y*_5,1_ and *y*_5,2_ must have their strengths changed in an identical way by STDP. This means that the connection strengths from neurons in the center stem to those on the left and right arm of the T-maze are equal, resulting in no preference for either path. To show that this is indeed the case in the full spiking network, we calculate the bias of connections strengths from neurons in the center stem to all other neurons (see Materials and methods); Fig 5a shows that this bias value is small for neurons in the center stem, indicating that changes in coupling strength for these neurons are symmetric. These results indicate that the changes of coupling strengths due to STDP can learn the paths of the model rat, but that these changes have not broken the symmetry between the two paths in the network.

The changes of coupling strengths between the cue neurons and the neurons within the 2D spiking neural circuit, however, are asymmetrical, with connections to one arm of the T-maze increased whilst those to the other arm are unchanged. As before, we consider a group of neurons which are activated sequentially by a moving rat, but we now examine the changes of the connection strengths from the cue neuron *C*2 to the neurons in the two-dimensional network (Fig 4c), with the cue neuron firing at the same time as the neuron *y*_4_. For this case, the interplay between STDP and the evoked firing sequence changes the coupling strengths in an asymmetrical way, as shown in Fig 4c; this occurs because for each training trial as *C*2 fires, the model rat is forced to travel along the right arm of the T-maze. As a result, the connection strengths from *C*2 to the neurons along the right arm of the T-maze are increased, whilst connections from *C*2 to the neurons along the left arm are unmodified by STDP and maintain their original values.

Likewise, the connections from *C*1 to the neurons along the left arm of the T-maze will have their strengths increased, whilst connections from *C*1 to the neurons along the right arm of the T-maze are not changed. On each successive training trial, this symmetry breaking becomes stronger as these differences in the connection strengths to the two different paths are magnified. We have verified that this mechanism indeed results in such asymmetric changes to the coupling strengths from the external cue neurons to the neurons within the 2-D spiking neural circuit (Fig 5b).

These changes in synaptic coupling strengths due to the interactions between the sequence evoked by the moving rat and STDP can be shown to eventually result in the formation of a forward-sweep IGS when the rat approaches the decision point. Suppose that the trained model rat is at the decision point (Fig 1), and generates a bump of activity corresponding to this location. As we have demonstrated above, the connections from the decision point to the nearby neurons along both arms of the T-maze have had their strength increased by STDP (Fig 5a). When an external cue is applied, the membrane potentials of the neurons along one side of the T-maze are increased more than on the other side, because the connections from the external cue are asymmetrical, as shown in Fig 5b. These increased membrane potentials cause these neurons to fire before those on the other arm, therefore leading to the formation of a spiking neural sequence sweeping away from the decision point of the T-maze towards the goal location. We note that without training, the asymmetry in the connection strengths does not exist, and the rat is unable to make a decision about which goal is correct given the cue.

In our model without STDP, the paths of the model rat cannot be learned and as a result the IGS cannot make a correct choice. To understand the contribution of synaptic scaling to the choice made by the neural circuit shown in Fig 3, we now study the behaviour of the network in the absence of synaptic scaling. We find that for the first 40 trials the proportion of correct responses when *C*2 is applied is almost identical in the model with and without synaptic scaling, as shown in Fig 3. However, after 40 trials, the synaptic behaviour of the two models is no longer similar. The major difference is that in the model without scaling, it becomes increasingly common for the neural sequence to split and travel along both paths. After 80 trials, this has become the dominant response; this behaviour is inconsistent with experimental results [6]. As described earlier, the strength of synapses along the direction of propagation of sequences are increased. As training trials occur with the goal on both the left and right arm of the T-maze, connections from neurons in the center stem to those on the left and right arm are both increased, as in Fig 4b. After sufficient training, the additional excitation generated from these synapses may be sufficient to cause a neuron on one of the arms of the T-maze to spike without any input from the external cue. As a result, the sequence splits and travels down both arms of the T-maze simultaneously. Synaptic scaling, however, causes coupling strengths to gradually return to their original values, effectively reducing the excitation received by neurons on the arms of the T-maze, and preventing the sequence from splitting and propagating along both arms of the T-maze. In addition, due to synaptic scaling, recent changes to synaptic strength have more impact on the current synaptic strength than those that occurred earlier in the training process. As illustrated in the later sections, this temporal property is crucial for the network to generate adaptive choices in response to time-varying and stochastic cue-goal associations.

### Recursive Bayesian inference and IGS

To study the general computational roles of the IGS that emerge from our spiking neural circuit model, we now consider the cases in which the cue-goal associations can be both probabilistic and time-varying. We introduce *g*_*n*_, which is the probability that on trial *n*, the goal location is on the right arm when *C*2 is the supplied cue. To maintain symmetry between *C*1 and *C*2, whilst ensuring that the number of left and right trials is the same, the probability that on the *n*th trial the goal location is on the left arm when *C*1 is the supplied cue is also *g*_*n*_; note that for the cue-goal association considered in the previous section, *g*_*n*_ = 1 for both *C*1 and *C*2.

**Fig 6:**
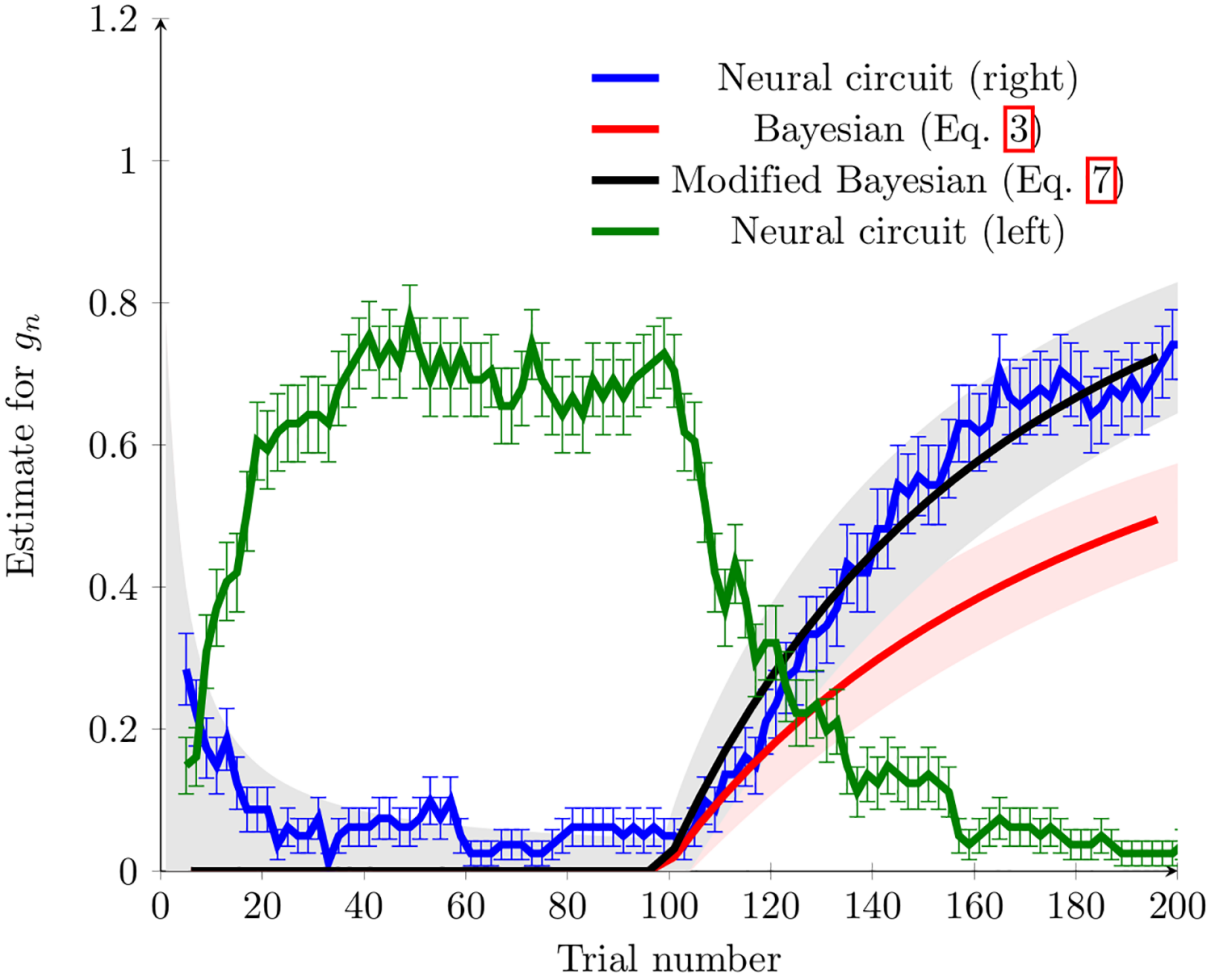
A comparison of estimation methods in the switching task in the T-maze. After 100 training trials, the association between cues and goals was switched. We note that the neural circuit adapts to this change, with rapid adaptation of the response rates to be more consistent with the new relationship between cue and goal. The neural circuit is shown by the blue curve, the simple Bayesian method (Eq 3) shown by the red curve, and the modified Bayesian method (Eq 7) shown by the black curve. The green curve shows the proportion of trials in which the propagating wave pattern travelled down the left arm of the T-maze, rather than the right arm.Alternatively, it follows from a simple symmetry argument that the green curve shows the proportion of trials in which the propagating wave pattern travelled down the right arm of the T-maze, but with the order training trials reversed. The fact that the green and blue curves are separated at trial 200 indicates that the neural circuit model is sensitive to the order of trials, as in both cases, 100 *L* and 100 *R* trials have occurred. For the neural circuit, error bars show the SEM, and for the Bayesian methods (Eqs 3 and 7) shading shows the 95% confidence interval. We note the responses from the neural circuit are similar to that given by Eq 7, and the estimate from Eq 3 is poor.

We first consider a simple case of time-varying cue-goal association in which an initial association between the cue and the goal is first learned and then switched. This switch takes the form of a step-like change in *g*_*n*_ after a certain number of trials; in this study, we have *g*_*n*_ = 0 when *n* ≤ 100, followed by *g*_*n*_ = 1 for *n* > 100. Such a switching change has been used to study the behaviour of rats in a T-maze goal-directed choice protocol [23], similar to ours. Fig 6 shows the correct response rate (blue line) when *C*2 is used as the test cue under this protocol. When we compare the results from the experimental study [23] to our modelling study, we find that significant similarities exist. In particular, after the switch of the cue-goal association, in both cases the correct response rate increases quickly. This increase then slows before a saturation level is reached. In our study this takes around 60 trials, whilst in [23], 40 training trials were necessary for all rats to learn the cue-goal relationship. The similarity between the correct response rate from the neural circuit model and the experimental data provides further evidence that our IGS-based model is able to respond to the switching change in the cued-choice task. Again, we find that the model without synaptic scaling cannot respond to this change, in particular, after 200 trials, responses almost exclusively consist of the non-physiological splitting behaviour and the correct response rate is very low.

It has been proposed that goal-directed decisions and planning can be understood as an implementation of probabilistic inference [21, 12]. We shall show how such probabilistic inference can be related to our IGS-based decision making model. In general, the problem of probabilistically estimating the value of *g*_*n*_ is an example of a hidden variable problem, since the variable of interest (i.e., *g*_*n*_) cannot be measured directly, but instead its value is estimated based on observations. In the context of the problem we are considering, these observations come from the training trials. We label the observation obtained on the *n*th training trial as *y*_*n*_, which may take one of two values, namely *L* and *R*, corresponding to the goal location being on the left and the right arm of the T-maze, respectively. We assume that *y*_*n*_ is a martingale, i.e., it is unaffected by the value of *y*_1_, …, *y*_*n*−1_, so that *p*(*y*_*n*_|*y*_1_, …, *y*_*n*−1_) = *p*(*y*_*n*_). The sequence of these observations is denoted by *Y*_*n*_ = *y*_1_, *y*_2_, …, *y*_*n*_. In a Bayesian context, to solve the hidden variable problem, we need to calculate the conditional posterior distribution function (pdf) *p*(*g*_*n*_|*Y*_*n*_). We now provide a brief overview of how this calculation can be performed. Using the notation we have outlined above, the conditional pdf can be calculated in the following way [24]:

$$\begin{array}{cc} p(g_{n}|Y_{n}) & =\frac{p\left(Y_{n}|g_{n}\right)p\left(g_{n}\right)}{p(Y_{n})}\\ & =\frac{p\left(y_{n},Y_{n-1}|g_{n}\right)p\left(g_{n}\right)}{p(y_{n},Y_{n-1})}\\ & =\frac{p\left(y_{n}|Y_{n-1},g_{n}\right)p\left(Y_{n-1}|g_{n}\right)p\left(g_{n}\right)}{p\left(y_{n}|Y_{n-1}\right)p\left(Y_{n-1}\right)}\\ & =\frac{p\left(y_{n}|Y_{n-1},g_{n}\right)p\left(g_{n}|Y_{n-1}\right)p\left(Y_{n-1}\right)p\left(g_{n}\right)}{p\left(y_{n}|Y_{n-1}\right)p\left(Y_{n-1}\right)p\left(g_{n}\right)}\\ & =\frac{p\left(y_{n}|g_{n}\right)p\left(g_{n}|Y_{n-1}\right)}{p(y_{n}|Y_{n-1})}, \end{array}$$ (2)

where *p*(*y*_*n*_|*g*_*n*_) is the likelihood function, *p*(*g*_*n*_|*Y*_*n*−1_) is the prior and *p*(*y*_*n*_|*Y*_*n*−1_) is a normalizing factor. Calculating *p*(*g*_*n*_|*Y*_*n*_) gives the conditional probability density function for the goal being located on the right arm when *C*2 is applied, for the sequence of observations *Y*_*n*_. Assuming that *g*_*n*_ is constant, i.e. *g*_*n*_ = *g*_*n*−1_, Eq 2 can be simplified to:

$$\begin{array}{c} p\left(g_{n}|Y_{n}\right)=\beta_{n}p\left(y_{n}|g_{n}\right)p\left(g_{n-1}|Y_{n-1}\right), \end{array}$$ (3)

where *β*_*n*_ is an appropriate normalization constant which is independent of *g*_*n*_. This recursive formula for the posterior pdf treats the posterior pdf from the previous trial as a new prior, which is then updated by the observation on the current trial. To complete this formulation, we require both the initial prior and the likelihood function *p*(*y*_*n*_|*g*_*n*_). We choose *p*(*g*_0_) = 1 as a simple uninformative prior. The likelihood function calculates the likelihood of the observation *L* or *R*, given a value of *g*_*n*_. The likelihood function is then given by:

$$\begin{array}{cc} p(L|g_{n}) & =g_{n},\\ p(R|g_{n}) & =1-g_{n}. \end{array}$$ (4)

From this formulation, it can be shown that [25]:

$$\begin{array}{c} p\left(g_{n}|Y_{n}\right)=\frac{(\alpha +\beta -1)!}{(\alpha -1)!(\beta -1)!}g_{n}^{\alpha -1}{\left(1-g_{n}\right)}^{\beta -1}, \end{array}$$ (5)

where *α* is the number of *L* observations and *β* is the number of *R* observations. To make a concrete choice for the goal location, we use a maximum likelihood estimator, which gives an estimate ${\hat{g}}_{n}$ for *g*_*n*_ by choosing the *g*_*n*_ that maximizes the value of *p*(*g*_*n*_|*Y*_*n*_). For the case *g*_*n*_ = *g*_*n*−1_, it can be shown that

$$\begin{array}{c} {\hat{g}}_{n}=\beta /\left(\alpha +\beta \right)=\beta /n. \end{array}$$ (6)

It is clear that as *n* increases, ${\hat{g}}_{n}$ will tend towards *g*_*n*_, at least in the case where *g*_*n*_ is constant.

We now compare the choices made using IGS in our neural circuit model with a recursive Bayesian inference approach outlined above. We find that estimated values of *g*_*n*_ based on Eq 3 do not match the true values; for example, the prediction ${\hat{g}}_{200}=0.5$ compares poorly to the true value of *g*_200_ = 1. However, our neural circuit generates a more accurate estimate of *g*_*n*_ (Fig 6).

To understand how this discrepancy between the result from this probabilistic inference and our neural circuit model occurs, we consider whether both models are sensitive to the order of the training trials. As we described in Eq 6, the estimate ${\hat{g}}_{n}$ made by recursive Bayesian inference depends only on the total number of trials with the goal location on the left and right arm, and is independent of their temporal order. In contrast, we have found that our neural circuit model is sensitive to the order of the training trials; that is, if we reverse the order of the training trials (i.e. 100 *R*, which is followed by 100 *L*), the resulting estimate for *g*_*n*_ on the 200th trial is significantly different than the original case (i.e. 100 *L*, which is followed by 100 *R*), as shown in Fig 6. This sensitivity to the order of training trials in our spiking neural circuit happens because synaptic connection strengths learned by STDP can be gradually reset by synaptic scaling. This gradual resetting process means that more recent trials have a more significant effect on the coupling strengths than those in the distant past.

As accurate predictions for a changing *g*_*n*_ require sensitivity to the order of the training trials, we propose an extension to Eq 3; inspired by the effect of synaptic scaling on synaptic coupling strengths, we incorporate a similar resetting mechanism to Eq 3 and obtain the following:

$$\begin{array}{c} p\left(g_{n}|Y_{n}\right)=\beta_{n}p\left(y_{n}|g_{n}\right)p{\left(g_{n}|Y_{n-1}\right)}^{\alpha }, \end{array}$$ (7)

where 0 < *α* < 1 is a parameter that controls the contribution of the previous probabilistic estimate for *g*_*n*_ (i.e. *p*(*g*_*n*_|*Y*_*n*−1_)) to the new one (i.e. *p*(*g*_*n*_|*Y*_*n*_)). Expanding this formula, it becomes clear how this change is sensitive to the order of trials, similar to the effect of synaptic scaling:

$$\begin{array}{c} p\left(g_{n}|Y_{n}\right)=\beta_{n}p\left(y_{n}|g_{n}\right)p{\left(y_{n-1}|g_{n}\right)}^{\alpha }p{\left(y_{n-2}|g_{n}\right)}^{2\alpha }\ldots p{\left(y_{0}|g_{n}\right)}^{n\alpha }. \end{array}$$ (8)

Eq 8 indicates that *p*(*g*_*n*_|*Y*_*n*_) can be viewed as a product of the likelihood functions from the previous trials i.e., *p*(*y*_*n*−1_|*g*_*n*_)^*α*^ for the *n* − 1th trial, *p*(*y*_*n*−2_|*g*_*n*_)^2*α*^ for the *n* − 2th trial, etc. As *α* < 1, we have *p*(*y*_*n*_|*g*_*n*_) < *p*(*y*_*n*_|*g*_*n*_)^*α*^ < *p*(*y*_*n*_|*g*_*n*_)^2*α*^ < ⋯ < 1. This relationship indicates that more recent trials have a more significant contribution to the estimate of the current value of *g*_*n*_; this is necessary for capturing the temporal order effect of responding to the switching change in the cued-choice task. We find that by choosing an appropriate value of *α* (*α* = 0.99 in this case), the estimated value of *g*_*n*_ calculated using a maximum likelihood method from the modified recursive Bayesian inference method indeed matches that generated from our spiking neural circuit and the true value of *g*_*n*_ (Fig 6).

### Adaptive spatial decision making by using IGS

We have shown that our neural circuit model with IGS is able to adaptively respond to a switch in the cue-goal association, and that this behaviour is well approximated by a simple approach based on probabilistic inference. Now, we demonstrate that our IGS-based model can make real-time choices when *g*_*n*_ changes randomly, and that the performance of our model is close to the optimal choices implemented by a Kalman filter.

We first describe a Kalman filter in the context of our problem and then compare its performance with that from our IGS-based model. The basic operation of the Kalman filter can be understood as follows: the Kalman filter contains some internal state which incorporates the history of past measurements, each new observation is then incorporated into this internal state and used to make a prediction of the hidden variable; for a detailed discussion see [24]. The 1-dimensional Kalman filter for estimating *g*_*n*_ is given by the following set of equations:

$${\hat{g}}_{n+1}\;=\;\left(1-K\left(n\right)\right){\hat{g}}_{n}+K\left(n\right)y_{n},$$ (9)

$$\Sigma_{n+1}\;=\;{\left(1-K\left(n\right)\right)}^{2}\Sigma_{n}+K{\left(n\right)}^{2}Q+Z,$$ (10)

$$K\left(n\right)\;=\;\frac{\Sigma_{n}}{\Sigma_{n}+Q},$$ (11)

where ${\hat{g}}_{n+1}$ is the estimate of the hidden variable, i.e. the estimate for the current value of *g*_*n*+1_, *y*_*n*_ is the *n*th measurement as defined above, *Q* is the covariance of the measurement error, *Z* is the covariance of the variation in *g*_*n*_, *Σ*_*n*_ is the estimate of the covariance of ${\hat{g}}_{n+1}$ and *K*(*n*) is the Kalman gain. In several cases, it can be shown that the estimate ${\hat{g}}_{n+1}$ given by the Kalman filter is optimal. One of these cases is the Gaussian random walk, where the dynamics of the hidden variable and the measurement process are given by the following equations:

$$\begin{array}{cc} g_{n+1} & =g_{n}+\xi_{1}\left(n\right),\\ y_{n} & =g_{n}+\xi_{2}\left(n\right), \end{array}$$ (12)

where *ξ*_1_(*n*) and *ξ*_2_(*n*) are independent zero-mean Gaussian noise processes, with variance *Q* and *Z*, respectively. In the Kalman filter, the Kalman gain, *K*(*n*), controls how much each individual observation, *y*_*n*_ affects the internal state of the Kalman filter. If each individual observation is unreliable due to noise, i.e., *Z* is large, the Kalman gain is small and it takes many trials for the Kalman filter to change its estimate of *g*_*n*_. On the other hand, if each observation is accurate, i.e., *Z* is small, the Kalman gain is large and the Kalman filter will quickly change its estimate. In our study, to be consistent with the Gaussian random walk, we use *g*_*n*_ given by Eq 12 and *g*_0_ = 1/2. As our neural circuit adapts slowly to changes in *g*_*n*_, it is necessary that *Q* is small; we have used *Q* = 2 × 10^−5^. Satisfying the condition on *y*_*n*_ is more complex, to this end we must verify that the measurement error, *g*_*n*_ − *y*_*n*_ has a Gaussian distribution with mean 0. Numerically, we have found that the distribution of measurement errors is well approximated by a Gaussian distribution. As a result, we have shown that when *g*_*n*_ is given by Eq 12, the requirements for the Kalman filter to produce optimal estimates of *g*_*n*_ have been satisfied.

**Fig 7:**
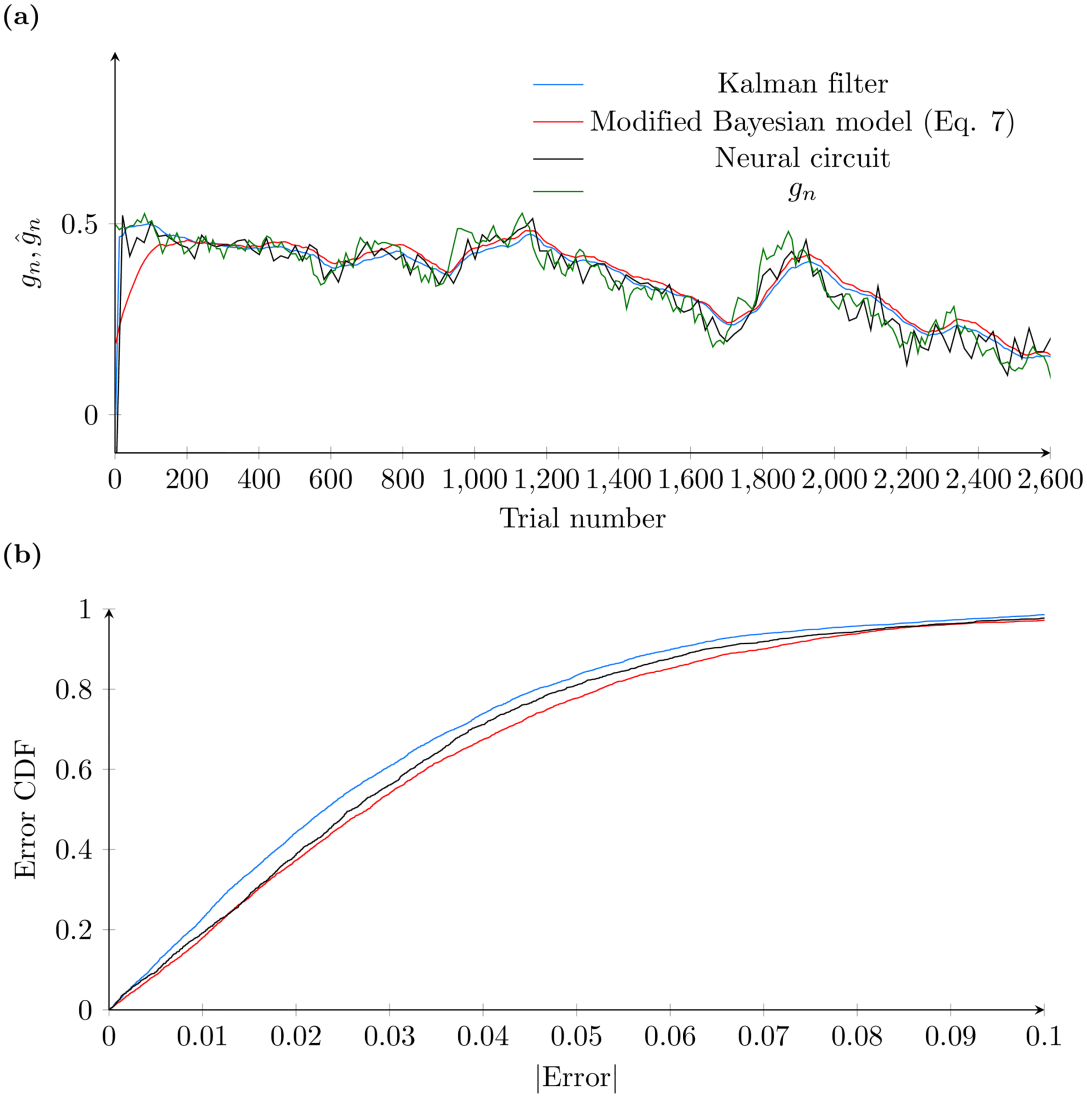
Comparison of real-time decision making performance by the different models. (a) Direct comparison of the models to the true values, given by *g*_*n*_. To enable a direct comparison a linear map has been applied to the result from the spiking neural circuit model and the value of *α* used in the Bayesian model (Eq 7) has been chosen by optimization as described in the main text. The green curve is for the true value of *g*_*n*_, the light blue curve is for the output from the Kalman filter, the red curve is for the Bayesian model and the black curve is for the neural circuit model. (b) Cumulative distribution functions for the errors of the various methods, colors as in (a). The error distributions are similar for all three models, although the Kalman filter has the best performance.

As noted earlier, the neural circuit estimates of *g*_*n*_ saturate at a level below 1, even when *g*_*n*_ = 1, as shown in Fig 3. To allow for a direct comparison with the Kalman filter, we thus use a linear map that scales the estimate of *g*_*n*_ to match the scale of the output of the Kalman filter; namely, we use ${\hat{g}}_{n}^{\prime }=a{\hat{g}}_{n}+b$, where ${\hat{g}}_{n}$ is the proportion of correct responses on the *n*th trial from our network model, with *a* and *b* chosen to minimize the mean squared difference between *g*(*n*) and ${\hat{g}}_{n}$. Fig 7a shows the estimated values of *g*_*n*_ from the spiking neural circuit, the modified recursive Bayesian inference procedure described by Eq 7 and the Kalman filter. We note that the parameters of the Kalman filter must be tuned to match both the measurement noise and the noise in the random walk, as they are crucial parameters describing the behaviour of the Kalman filter. The parameters of the neural circuit, however, are identical to those used in the earlier sections. It is apparent that all methods closely follow the changes in *g*_*n*_ when it undergoes a Gaussian random walk.

To enable a more quantitative comparison between the models, we calculate a cumulative distribution function for the absolute error, i.e. $|g_{n}-{\hat{g}}_{n}|$; as shown in Fig 7b, the neural circuit model and Eq 7 have a similar accuracy to the Kalman filter, specifically, the estimate for *g*_*n*_ from the neural circuit was within 3% of the correct value on 50% of trials, within 5% on 80% of trials and within 6% on 90% of trials. The fact that our neural circuit model achieves accuracy comparable to the Kalman filter suggests that our neural circuit model is indeed capable of making a real time estimate of *g*_*n*_.

It may be possible to further increase accuracy by incorporating a reward signal. For example, in [26], a synaptic update rule was obtained from the theoretical consideration of maximizing the probability of receiving a reward event, but in our study we use biologically plausible plasticity mechanisms (i.e., STDP and synaptic scaling). Additionally, in our model we study a general goal-location decision task by considering time varying cases of cue-goal association, which was not studied in [26].

### Changes of coupling strengths and time-varying cue-goal association

We now illustrate how the combined synaptic plasticity rules (i.e. STDP and synaptic scaling) enable the changes to coupling strengths to encode time-varying cue-goal associations; this mechanism underlies the adaptive choice results reported above.

In the previous sections, we showed that connections from the external cue to neurons on the left or right arms of the T-maze such as *y*_5,2_ (Fig 4c) are crucial for determining which path the neural sequence would choose to travel down. We now consider the dynamics of the synapse connecting *C*2 to neuron *y*_5,2_, which are driven by both STDP and synaptic scaling, in order to understand the choice mechanism within our neural circuit, although our analysis does not crucially depend on the precise location of the neuron. In particular, the increase in coupling strength due to STDP in a single trial is *A*_+_ exp(−Δ*t*_5_/*τ*_+_) (Eq 22). However, it is not necessarily true that on every training trial, the model rat travels along the right arm of the maze, where the sample neuron is located. In fact, the probability of this event is given by *g*_*n*_, by its definition. It follows that the average increase in coupling strength per trial is *A*_+_*g*_*n*_ exp(−Δ*t*_5_/*τ*_+_).

As we described in the Materials and Methods section, synaptic scaling causes a gradual resetting in coupling strengths towards their initial values. It follows that over the time period of a learning trial, some fraction of the changes in coupling strength caused by STDP will be lost by this resetting process. We can find the value of this loss as follows: if at the start of the trial, the change in the connection strength from its initial value is Δ*W*; at the end of the trial, synaptic scaling will have reduced it to *r*Δ*W* (see Materials and methods). This decay occurs on every trial, regardless of whether the rat travels down the left or right arm of the T-maze. This gives a recursive equation for the change in coupling strength of the synapse from the external cue to our sample neuron after the *n*th trial, Δ*W*_*n*_, as follows:

$$\begin{array}{c} \Delta W_{n}=A_{+}g_{n}\exp\left(-\Delta t_{5}/\tau_{+}\right)+r\Delta W_{n-1}. \end{array}$$ (13)

Analyzing this equation directly is difficult, so we consider a simplification based on the assumption that *g*_*n*_ is either constant or varying slowly, as in Figs 3 and 7. To this end, rather than using the value of *g*_*n*_, we use ${\bar{g}}_{n}=\sum_{i=1}^{N}g_{n-i}/N$ where *N* is chosen depending on both the dynamics of *g*_*n*_ and the neural circuit. We have chosen a value of *N* = 50 based on the time it takes for the correct response rate to saturate, as shown in Fig 3. By setting Δ*W*_*n*_ = Δ*W*_*n*−1_ = Δ*W*_*ss*_, we can find the value of the steady state coupling strength change Δ*W*_*ss*_:

$$\left(1-r\right)\Delta W_{ss}\;={\bar{g}}_{n}A_{+}\text{exp}\left(-\Delta t_{5}/\tau_{+}\right).$$ (14)

This then gives an explicit description of Δ*W*_*ss*_:

$$\begin{array}{c} \Delta W_{ss}={\bar{g}}_{n}A_{+}\frac{\exp(-\Delta t_{5}/\tau_{+})}{1-r}. \end{array}$$ (15)

As all variables other than ${\bar{g}}_{n}$ are fixed parameters of the model, we have:

$$\begin{array}{c} \Delta W_{ss}=F{\bar{g}}_{n}, \end{array}$$ (16)

where $F=\frac{A_{+}\exp\left(-\Delta t_{5}/\tau_{+}\right)}{1-r}$.

**Fig 8:**
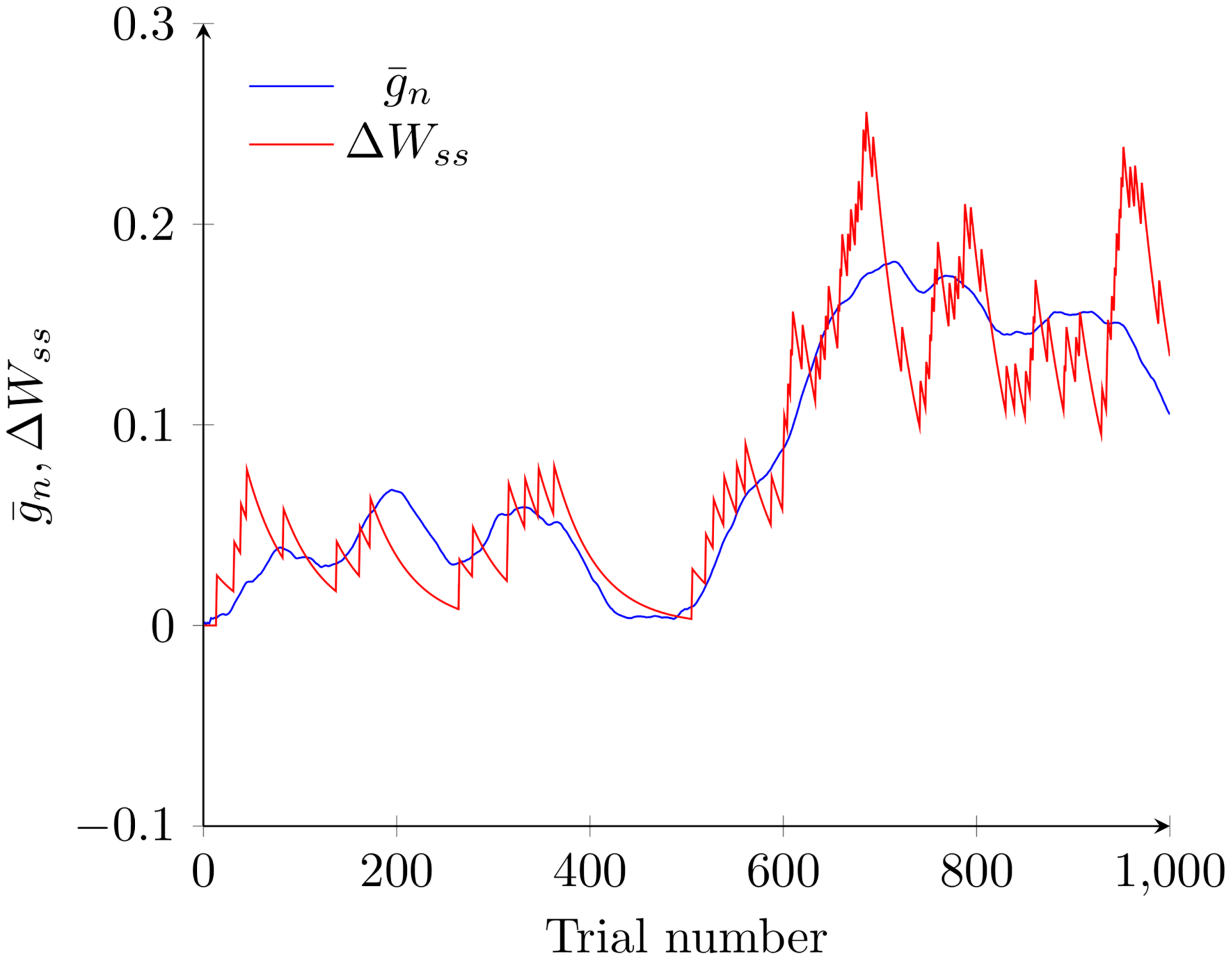
Coupling strength values closely trace ${\bar{g}}_{n}$. We compare Δ*W*_*ss*_ and ${\bar{g}}_{n}$ to verify that they follow each other closely. To enable this comparison, they are normalized so that they have identical means. All relevant parameters for both the dynamics of *g*_*n*_ and Δ*W*_*ss*_ are consistent with Fig 7.

It is clear from Eq 16 that the change in coupling strength is proportional to ${\bar{g}}_{n}$; this point is further illustrated in Fig 8 in which Δ*W*_*ss*_ and ${\bar{g}}_{n}$ are compared. These results thus indicate how the synaptic strengths mediated by the combination of STDP and synaptic scaling to some extent are able to adaptively trace the time-varying *g*_*n*_. As the synaptic strengths are the neural basis for forming the IGS, as shown above, our model is able to respond correctly even when *g*_*n*_ is changing (Fig 7).

## Discussion

In this study, we have developed a spiking neural circuit to study the formation mechanisms of forward sweeping, internally generated sequences and their function in goal-directed spatial decision making. We have demonstrated that in our spiking neural circuit, the interplay between the network dynamics and the combined synaptic dynamics of STDP and synaptic scaling is essential for the formation of such neural sequences. Our model can capture the salient properties of these sequences and yields behavioural performance comparable to data obtained from behaving animals [6]. In addition, we have demonstrated that STDP when complemented by slower synaptic scaling enables the neural sequences generated in the network model to adaptively respond to changing cue-goal associations.

Our model of spatial decision making shares a resemblance with other spreading-activation or propagating wave-based models for path planning [27, 28, 29] in that propagating sequential activity is a key feature used for making choices. However, unlike these models, but consistent with experimental data, there is no backwards diffusion of activity from the goal to the current state during the learning or planning process. Furthermore, by implementing goal directed decision making in probabilistic terms, our model also extends to the cases in which uncertainty in the cue-goal association exists, which has not been generally addressed by previous spreading activation models. Goal directed learning has previously been studied in spiking networks [26, 30, 31, 32]. However in these studies, goal-directed decision making with time-varying cue-goal association has not been considered, and how this can be implemented by biologically realistic synaptic mechanisms has not been addressed.

As we have demonstrated, STDP causes the coupling strengths along the direction of the model animal’s movement to increase and those in the opposite direction to decrease on each training trial. Such positive feedback effects from STDP alone would eventually result in the saturation of synaptic coupling strengths, so that the formed neural sequence propagates towards all possible goals, rather than toward one goal at a time. As a result, the spiking network neither can give rise to correct choice rates as reported in [6], nor make flexible, time-varying choices of goal locations. The homeostatic synaptic scaling rule, however, plays a role in preventing saturation of synaptic coupling strengths as the training process proceeds.In our model, this scaling process happens at a temporal scale which is around 350 times longer than that of STDP, therefore enabling the network to intrinsically possess distinct temporal scales. We have found that the behaviour of our model is not dependent on the precise values of the time constants chosen, as long as the timescale of STDP is shorter than that of a single learning trial and the timescale of synaptic scaling is much longer than both of these. In addition, changing the size of the neural circuit and T-maze does not significantly change our results; we have found that similar results can be found with T-mazes which are either twice or half the size of the T-maze that we have used in this study.

A combination of plasticity rules with separate temporal scales enables the network to maintain the trace of the moving paths of the model rat during the training process, and it provides the needed flexibility to allow the network to generate time-varying choices. Both STDP [33, 34, 35] and synaptic scaling [36] have been widely observed in the brain, and our model results show that the loss of either mechanism would impair the normal functions of the brain. In previous modelling studies, the combination of STDP and synaptic scaling has been mainly used to achieve specific network states, such as balanced states [37]. Our study, however, relates this combination and the resultant separation of time scales to network dynamics in terms of emergent IGS, and further to goal-directed spatial decision making. Other synaptic mechanisms such as short-term depression [38] and homeostatic synaptic plasticity described by the BCM rule [39] when combined with STDP may produce a similar behaviour, whilst also avoiding the saturation of coupling strength.

The IGS in our model capture the characteristic dynamics of IGS as observed in [6], including their forward propagation, and propagation toward one goal at a time rather than towards all goals simultaneously. Some previous modelling studies used spreading activation or propagating wave fronts to model path planning or goal directed decision making [27, 28, 29]; in these models, however, neural activity propagates backwards from the goal to the current location. Importantly, by reproducing animals’ behavioural responses, i.e., the correct decision rates as measured in [6], our modeling study directly demonstrates that such forward-sweeping sequences could be the neural substrate for implementing learning and implementing goal-directed decision making. In our study, this computational role of forward-sweeping IGS has been further illustrated by extending the deterministic cued-choice task studied in [6] to tasks with time-varying and probabilistic associations between cues and goals. This extension allows us to show that IGS-implemented functionality can be generally understood in terms of probabilistic inference. The general computational role of IGS revealed in our model is consistent with the proposal that IGS can implement probabilistic inference that optimizes goal acquisition for real time choice and learning [12, 21]. Our study thus provides a basis for further extensions considering reward learning and multiple choices.

In our model, when the probabilistic associations of the cues and goals undergoes a Gaussian random walk, such IGS-based decision making works in a near optimal way; the estimates are within 3% of the correct value for 50% of the time, largely comparable to those obtained by a Kalman filter that is optimal for this case. The Kalman filter has been well studied in relation to optimal decision making in prediction and motor control, however, neural representations of Kalman filters are largely unknown. By comparing our model with the working mechanism of the Kalman filter, we can understand how it is able to make accurate choices. In particular, in our IGS-based model for the spatial decision task, the interplay of spiking sequences and the combined synaptic rules lead to constant changes of synaptic coupling strengths. As we have demonstrated, these changes are proportional to the probability of cue-goal associations, as long as this probability varies slowly. Thus these changes to coupling strength can serve as the posterior for test trials, which can then be exploited by the sequences to make a choice. The ratio between the strength of STDP and synaptic scaling plays a similar role as the Kalman gain, as it controls how quickly new training trials are incorporated into the internal model. Specifically, STDP controls the strength of the trace left by the previous training trials, whilst synaptic scaling controls the speed with which they are reset to their original values. This ratio is fixed in our case, but Kalman gain is dynamical; this difference suggests that including other synaptic mechanisms such as meta-plasticity to the model may give rise to a dynamical ratio with multiple timescales [40], which may make the spiking model perform optimally like a Kalman filter. Nevertheless, our spiking neural circuit model suggests that the IGS and the combined synaptic plasticity rules are candidate neural implementations of a Kalman filter-based estimation of the changing cue-goal associations.

## Materials and methods

We consider a two dimensional, conductance-based integrate-and-fire neural network. In this 2D network, neurons are located at the integer intersections of a 200 × 200 grid, with inhibitory neurons located at points where both coordinates are even and excitatory neurons located at all other integer coordinates. The network has 30,000 excitatory neurons and 10,000 inhibitory neurons (i.e. 75% excitatory and 25% inhibitory neurons). Every neuron within the network connects with nearby neurons within 25 grid distance (arbitrary units). The 2D network has periodic boundary conditions, meaning that neurons on the edge can connect to neurons on the opposite edge to maintain an identical spatial arrangement of connectivity as the neurons in the middle of the network. There are also 2 cue neurons that connect to the neurons within the 2D network, with coupling weights chosen randomly from a distribution equivalent to the distribution of the weights within the 2D network, and with the same synaptic plasticity rules as in the 2D network. Code for this model is available at the following URL: https://github.com/BrainDynamicsUSYD/spikegrid.

We denote the membrane potential of a neuron at coordinates (*i*, *j*) at time *t* as *V*_*ij*_(*t*), with dynamics governed by the following equation:

$$\begin{array}{c} C\frac{d}{dt}V_{ij}\left(t\right)=-g_{L}\left[V_{ij}\left(t\right)-V_{L}\right]-g_{ij}^{E}\left(t\right)\left[V_{ij}\left(t\right)-V_{E}\right]-g_{ij}^{I}\left(t\right)\left[V_{ij}\left(t\right)-V_{I}\right], \end{array}$$ (17)

where the capacitance *C* = 1 *μ*Fcm^−2^, the leak conductance *g*_*L*_ = 50 *μ*Scm^−2^ and the reversal potentials are *V*_*L*_ = −70 mV, *V*_*E*_ = 0 mV and *V*_*I*_ = −80 mV for the leak, excitatory and inhibitory conductances, respectively. When the membrane potential of a neuron reaches a threshold *V*_*th*_ = −55 mV, a spike is generated. Subsequently, the neuron remains at the reset potential *V*_*R*_ = −70 mV for a period of time corresponding to the refractory state *τ*_*ref*_ = 5 ms. The dynamics of a synaptic conductance *g*^λ^ are given by:

$$\begin{array}{c} g_{ij}^{\lambda }\left(t\right)=F^{\lambda }+\sum_{i^{\prime }j^{\prime }}K_{ij,i^{\prime }j^{\prime }}^{\lambda }\left(t\right)\sum_{l}G^{\lambda }\left(t-T_{i^{\prime }j^{\prime }}^{l}\right), \end{array}$$ (18)

where λ is used to denote the two types of neurons, excitatory and inhibitory, represented by *E* and *I* respectively, and $T_{i^{\prime }j^{\prime }}^{l}$ is the time of the *l*-th spike emitted by the afferent neuron located at (*i*′, *j*′). A constant inhibitory input is supplied to ensure that no spontaneous firing of neurons occurs. This is achieved using *F*^*I*^ = 15 *μ*Scm^−2^ whilst *F*^*E*^ = 0 *μ*Scm^−2^. The time course of the post-synaptic conductance is given by

$$\begin{array}{c} G^{\lambda }\left(t\right)=\frac{\exp\left(-t/\tau_{d}^{\lambda }\right)-\exp\left(-t/\tau_{r}^{\lambda }\right)}{\tau_{d}^{\lambda }-\tau_{r}^{\lambda }}. \end{array}$$ (19)

Here we set $\tau_{d}^{I}=\tau_{d}^{E}=2.0$ ms, $\tau_{r}^{I}=\tau_{r}^{E}=0.5$ ms,. The denominator ensures that the total post-synaptic conductance is normalized such that $\int_{0}^{\infty }G^{\lambda }\left(t\right)\;dt=1$.

The coupling functions $K_{ij,i^{\prime }j^{\prime }}^{\lambda }$ have two parts, a static component $W_{ij,i^{\prime }j^{\prime }}^{\lambda }$ and a dynamic component, Δ*W*_*ij*,*i*′*j*′_(*t*) governed by STDP and synaptic scaling. The static coupling strength between two neurons located at (*i*, *j*) and (*i*′, *j*′) is given by:

$$\begin{array}{c} W_{ij,i^{\prime }j^{\prime }}^{\lambda }= \end{array}\{\begin{array}{ll} C^{\lambda }e^{-d_{ij,i\prime j\prime }^{2}/{\left(d^{\lambda }\right)}^{2}} & \text{if }d_{ij,i\prime j\prime }\leq \;D,\\ 0 & \text{if }d_{ij,i\prime j\prime }>\;D, \end{array}$$ (20)

where *d*_*ij*,*i*′*j*′_ is the Euclidean distance between the neurons on a square lattice, taking into account the periodic boundary conditions (i.e., neurons near the edge of the grid are coupled to those on the opposite side of the grid), and *C*^*E*^ = 5 × 10^−7^ and *C*^*I*^ = 2.4 × 10^−7^. We have chosen $d^{E}=\sqrt{20}$, $d^{I}=\sqrt{90}$ and *D* = 25. As a result, each neuron is connected to 1470 excitatory neurons and 490 inhibitory neurons. As in other models for path planning [41, 19], in our model the position of the neuron is used as a stand-in for the position of the neuron’s place field. In this case, a localized activity pattern is present surrounding the actual position of the model rat. Such a regular arrangement of place fields, as often used in modelling studies, provides a simple way to study the mechanisms of moving-path related planning and decision making tasks. The size of place fields has the same size as the excitatory coupling range, which is 25 grid points. The T-maze environment has a width of 11 grid points, which is slightly smaller than the place field. A trip through the T-maze takes approximately 100ms, and trials are separated by 150 ms. Note that our results are not sensitive to these values; for instance, if we change the trial separation time to 200 ms, we can obtain similar results.

The dynamic component of coupling comes from plastic synapses modulated by STDP and synaptic scaling. We incorporate these plasticity mechanisms for all synapses. The form of the change in coupling strength is identical for excitatory and inhibitory synapses and is given by:

$$\Delta W_{ij,i^{\prime }j^{\prime }}(t)=\{\begin{array}{ll} \sum_{t_{ij}^{k}<t}\sum_{t_{i^{\prime }j^{\prime }}^{l}<t}H\left(t_{ij}^{k},t_{i^{\prime }j^{\prime }}^{l}\right)+\sum_{t^{\prime }<t}S(\Delta W_{ij,i^{\prime }j^{\prime }}(t^{\prime })) & \text{if }d_{ij,i^{\prime }j^{\prime }}\leq D,\\ 0 & \text{if }d_{ij,i^{\prime }j^{\prime }}>D, \end{array}$$ (21)

where $t_{ij}^{k}$ is the *k*th firing time of the neuron located at (*i*, *j*) and $t_{i^{\prime }j^{\prime }}^{l}$ is the *l*th firing time of the neuron located at (*i*′, *j*′) and *S*(Δ*W*_*ij*,*i*′*j*_(*t*′)) is the contribution from synaptic scaling, which we discuss later. $H(t_{ij}^{k},t_{i^{\prime }j^{\prime }}^{l})$ is the STDP window function:

$$H\left(t_{ij}^{k},t_{i^{\prime }j^{\prime }}^{l}\right)=\{\begin{array}{ll} A_{+}\text{exp}(-\Delta t/\tau_{+}) & \text{if }\Delta t>0,\\ -A_{-}\text{exp}(-\Delta t/\tau_{-}) & \text{if }\Delta t<0,\\ 0 & \text{if }\Delta t=0,\\ 0 & \text{if }|\Delta t|>5\tau , \end{array}$$ (22)

where $\Delta t=t_{ij}^{k}-t_{i^{\prime }j^{\prime }}^{l}$ is the time difference between the two spikes, *τ*_+_ and *τ*_−_ are the timescales for potentiation and depression respectively; *A*_+_ and *A*_−_ are the magnitude of potentiation and depression, respectively. In our study, *τ*_+_ = *τ*_−_ = 20 ms and *A*_+_ = *A*_−_ = 1.2 × 10^−9^; these values are consistent with experimental data [33, 42].

Synaptic scaling has been often modelled by using a rule which changes coupling strengths by an amount Δ*W* such as the following [43]:

$$\begin{array}{c} \Delta W=\epsilon (f-f^{*})W, \end{array}$$ (23)

where *f* is the current firing rate, f* is a target firing rate, *W* is the current synaptic coupling strength and *ϵ* is a constant which controls the speed with which synaptic scaling acts. We use an adaptation of this rule in our model in which the synapses are modulated by STDP and firing rates do not change significantly. Crucially we seek to maintain the ability of synaptic scaling to scale coupling strengths both up and down, as commonly observed in experimental studies [36, 35, 44]. We allow synaptic scaling to scale coupling strengths by making changes proportional to Δ*W*_*ij*,*i*′*j*′_(*t*) as follows:

$$\begin{array}{c} S\left(\Delta W_{ij,i^{\prime }j^{\prime }}\left(t\right)\right)=C\Delta W_{ij,i^{\prime }j^{\prime }}\left(t\right), \end{array}$$ (24)

where Δ*W*_*ij*,*i*′*j*′_(*t*), caused by plasticity (Eq 21), can be negative or positive, so can *S*(Δ*W*_*ij*,*i*′*j*′_(*t*)). The constant *C* is chosen so that in the absence of any firing, coupling strengths reset toward their original values with a characteristic time scale of 7 seconds. For simulation efficiency, we only calculate *S* once every 100 time steps. We have found that the behaviour of our model is not dependent on the precise values of the time constants chosen, as long as the timescale of STDP is shorter than that of a single learning trial and that the timescale of synaptic scaling is much longer than both of these.

Even with these values chosen from experimental data, pathological behaviour can be observed, in which coupling strength changes caused by STDP can create unbounded positive feedback loops. As synaptic scaling acts over a long time scale, it does not react fast enough to prevent this from happening. In our model, as in [45], we use a hard limit on connection strengths as follows:

$$\begin{array}{c} |W_{ij,i^{\prime }j^{\prime }}\left(t\right)|\leq L|W_{ij,i^{\prime }j^{\prime }}\left(0\right)|. \end{array}$$ (25)

We set *L* = 0.12 which prevents connection strengths from being modified too significantly from their initial values. Using the above formulation of STDP and synaptic scaling, we can calculate the time-dependent coupling term from Eq 21 as follows:

$$\begin{array}{c} K_{ij,i^{\prime }j^{\prime }}^{\lambda }\left(t\right)=W_{ij,i^{\prime }j^{\prime }}^{\lambda }+\Delta W_{ij,i^{\prime }j^{\prime }}\left(t\right). \end{array}$$ (26)

To incorporate external cues in our network model, without loss of generality, we let the external cue *Ci* have *N* = 40,000 synapses, with the post-synaptic neuron for each synapse chosen at random with replacement, i.e., an average of one connection per neuron in the 2D network. The initial strengths of these synapses are chosen at random so that the overall distribution of coupling strengths from the external cue is identical to the distribution of connections from a neuron in the 2D grid. Similarly, the synapses which connect the cue neurons to the neurons in the spatially extended neural circuit are mediated by both STDP and synaptic scaling, although as it is not possible for a feedback loop to form for a connection from the external cue, the limit on connection strengths given by Eq 25 is not applied.

### Bias in connection strengths

Analyzing the overall effect of plasticity is difficult due to the large number of synaptic connections in our model. To simplify this analysis, we construct a per-neuron measure which determines the bias in connection strengths emanating from that particular neuron. In particular we are interested in the directional bias of connection strengths to understand how decisions can be made in the cued-choice task. If an excitatory neuron in the center stem is more strongly coupled to neurons on the left arm of the T-maze, the sequence will be more likely to travel along the left arm of the T-maze. We construct the following measure of the bias in connection strengths inspired by a center of mass calculation as follows:

$$\begin{array}{c} B_{ij}\left(t\right)=|\sum_{i^{\prime }j^{\prime }}\left(i-i^{\prime }\right)W_{ij,i^{\prime }j^{\prime }}\left(t\right)| \end{array}$$ (27)

This measure will be small if connections from the neuron to the left and right have similar strength, and large otherwise, allowing simple analysis of any bias present in the network.
